# Supplementary material for: Cyclooxygenase-2 Blockade Is Crucial to Restore Natural Killer Cell Activity before Anti-CTLA-4 Therapy against High-Grade Serous Ovarian Cancer
Source: Cancers (Basel). 2023 Dec 22;16(1):80. doi: 10.3390/cancers16010080 (PMC10778357; doi:10.3390/cancers16010080)
Supplement: Supplementary file 1 [file cancers-16-00080-s001.zip › cancers-2757262-supplementary.pdf]

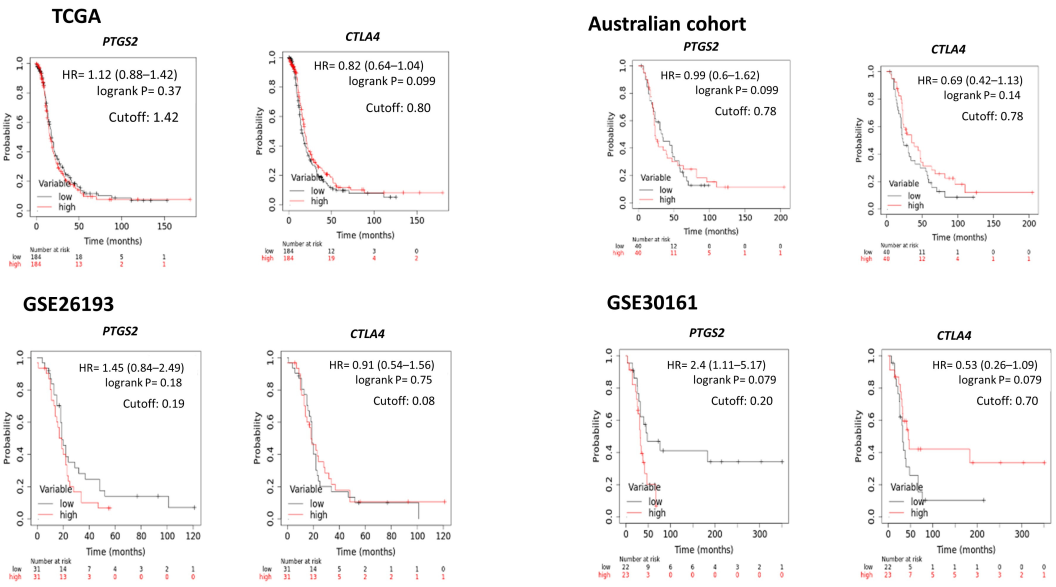

Figure S1. KMplot for *PTGS2* and *CTLA4* genes (defined by their median expression) in the four analyzed cohorts.

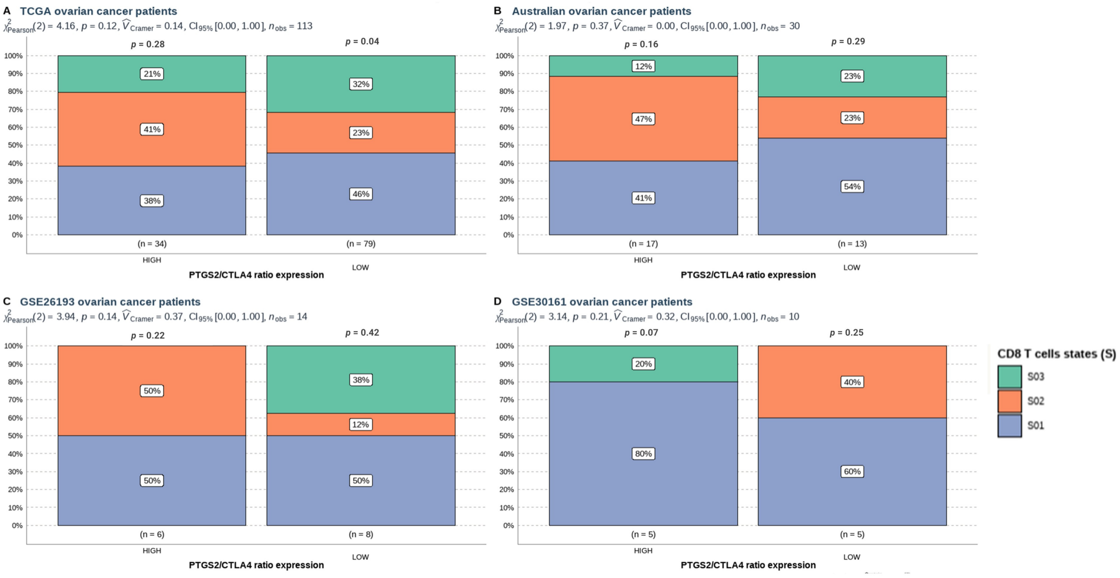

Figure S2. Prevalence comparison of CD8 T cell states (S) in (A) TCGA-GDC and (B) AOCS patients, (C) GSE26193, (D) GSE30161 ovarian patients' cohorts according to *PTGS2/CTLA4* ratio expression. Pearson's chi-squared test was performed through the "ggstatsplot" R package.

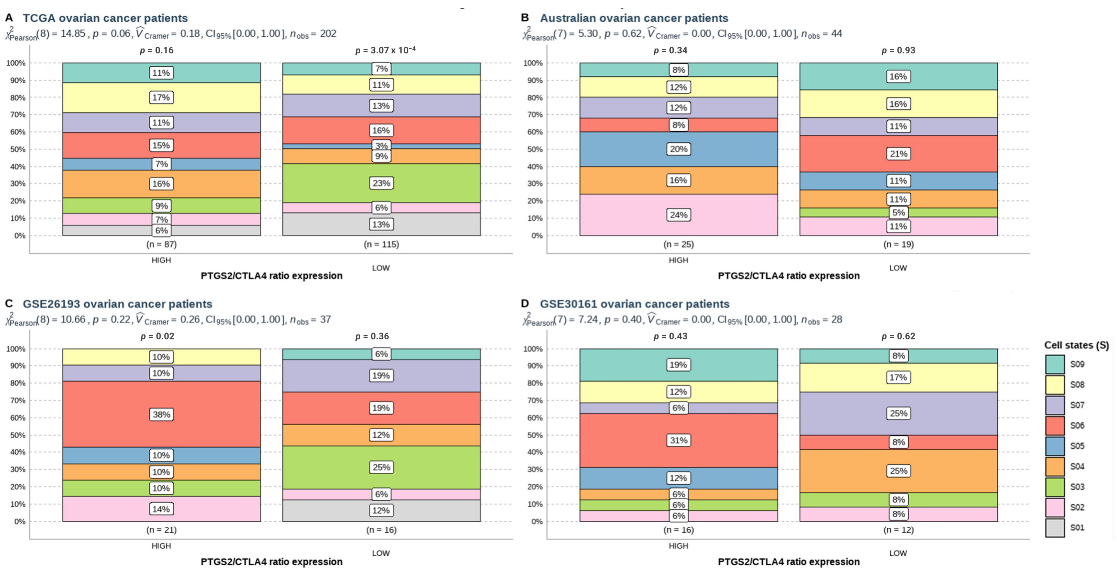

Figure S3. Prevalence comparison of monocytes/macrophages states (S) in (A) TCGA-GDC, (B) AOCS, (C) GSE26193, (D) GSE30161 ovarian patients' cohorts according to *PTGS2/CTLA4* ratio expression. Pearson's chi-squared test was performed through the "ggstatsplot" R package.

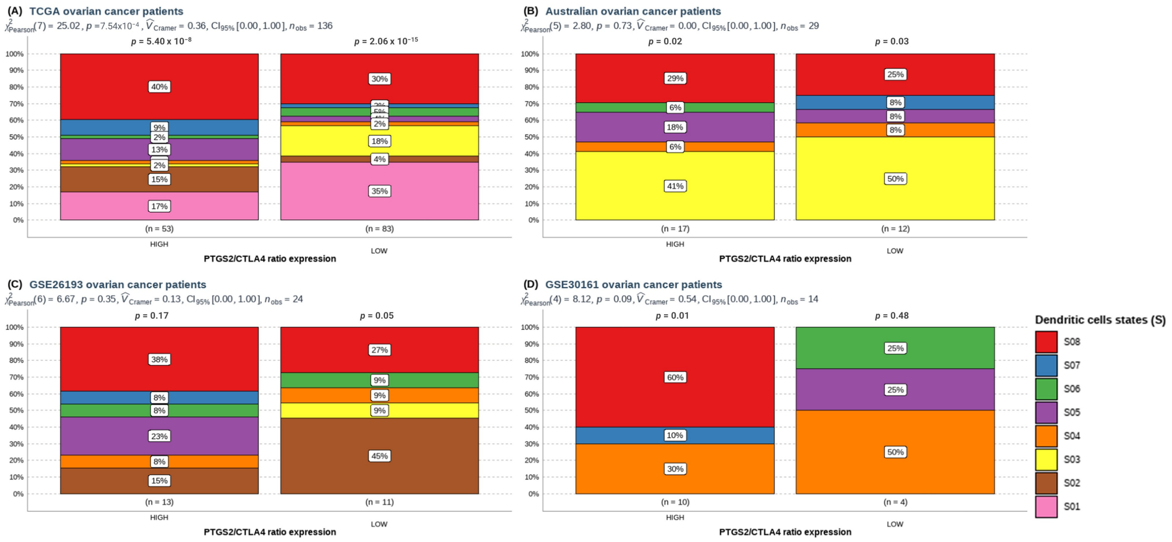

Figure S4. Prevalence comparison of dendritic cell states (S) in (A) TCGA-GDC, (B) AOCS, (C) GSE26193, (D) GSE30161 ovarian patients' cohorts according to *PTGS2/CTLA4* ratio expression. Pearson's chi-squared test was performed through the "ggstatsplot" R package.

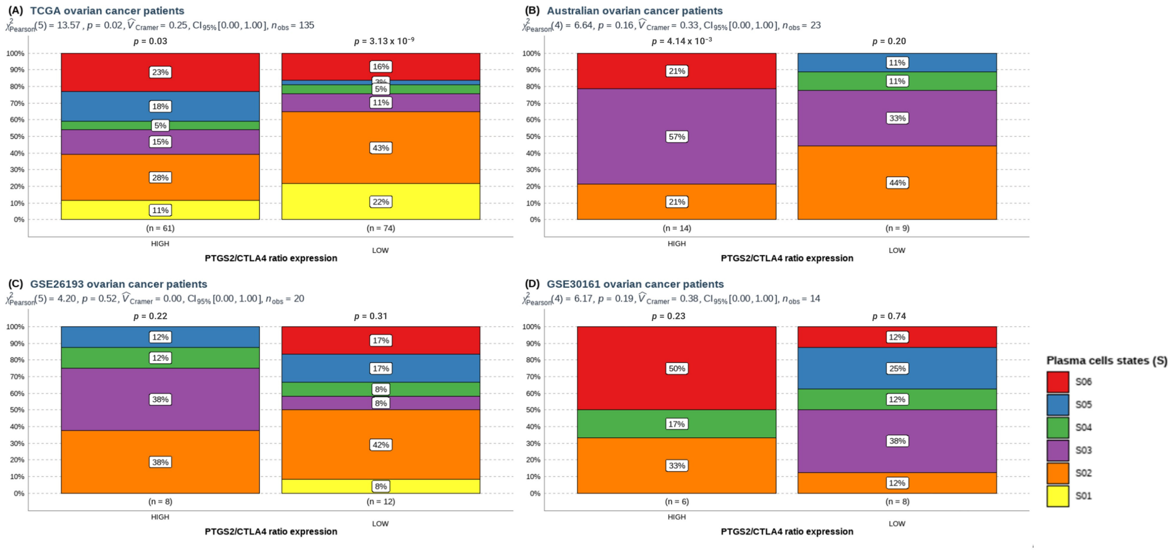

Figure S5. Prevalence comparison of plasma cell states (S) in (A) TCGA-GDC, (B) AOCS, (C) GSE26193, (D) GSE30161 ovarian patients' cohorts according to PTGS2/CTLA4 ratio expression. Pearson's chi-squared test was performed through the "ggstatsplot" R package.

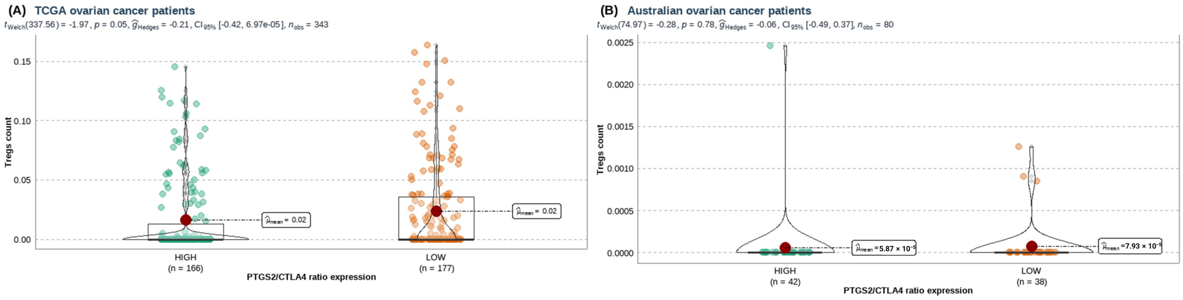

Figure S6. Comparison of MIXTURE's T regulatory cells (Tregs) estimation for (A) TCGA-GDC and (B) AOCS cohorts.

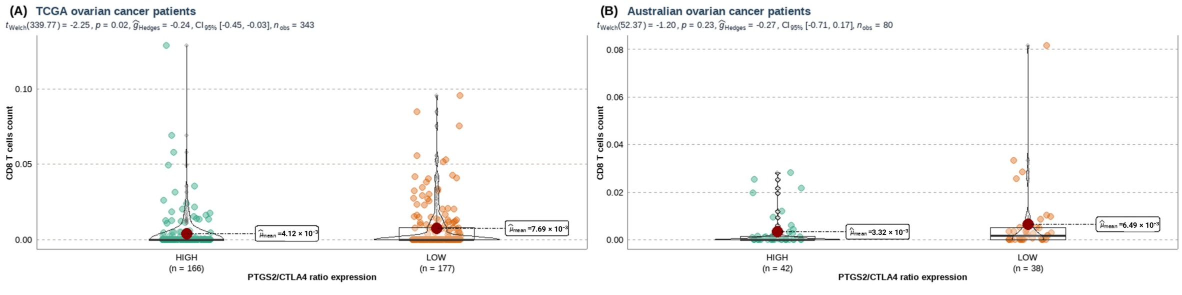

Figure S7. Comparison of MIXTURE's CD8 T cells estimation for (A) TCGA-GDC and (B) AOCS cohorts.

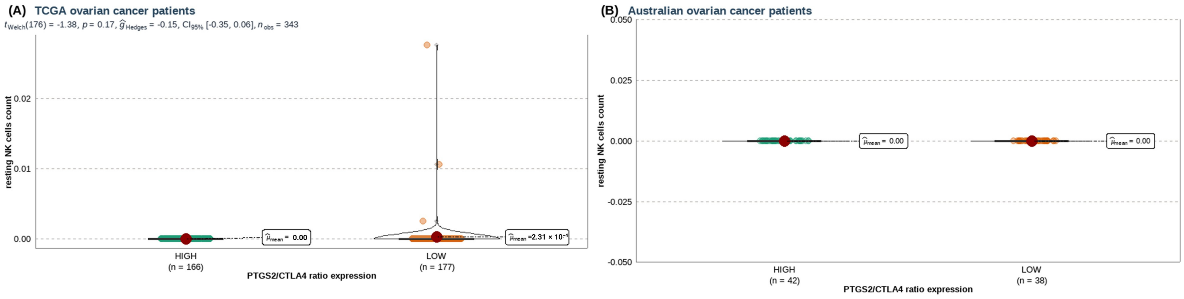

Figure S8. Comparison of MIXTURE's resting NK cells estimates for (A) TCGA-GDC and (B) AOCS cohorts.

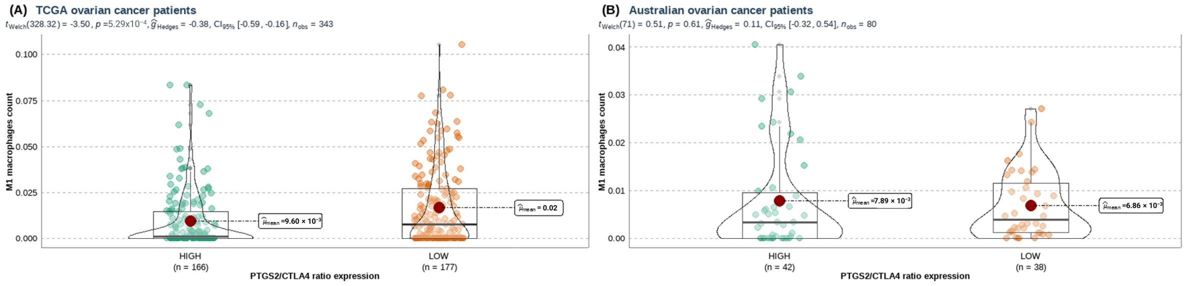

Figure S9. Comparison of MIXTURE's M1 macrophages estimates for (A) TCGA-GDC and (B) AOCS cohorts.

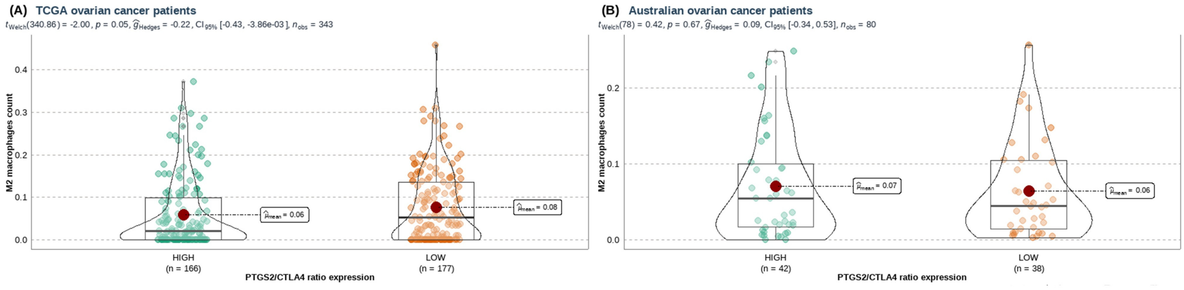

Figure S10. Comparison of MIXTURE's M2 macrophages estimation for (A) TCGA-GDC and (B) AOCS cohorts.

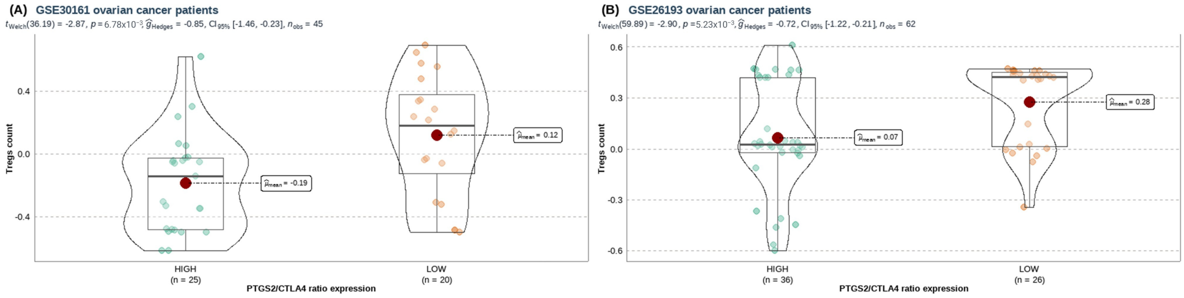

Figure S11. Comparison of GSVA score of Tregs estimation from Nieto's signature for (A) GSE30161 and (B) GSE26193 ovarian cancer cohorts.

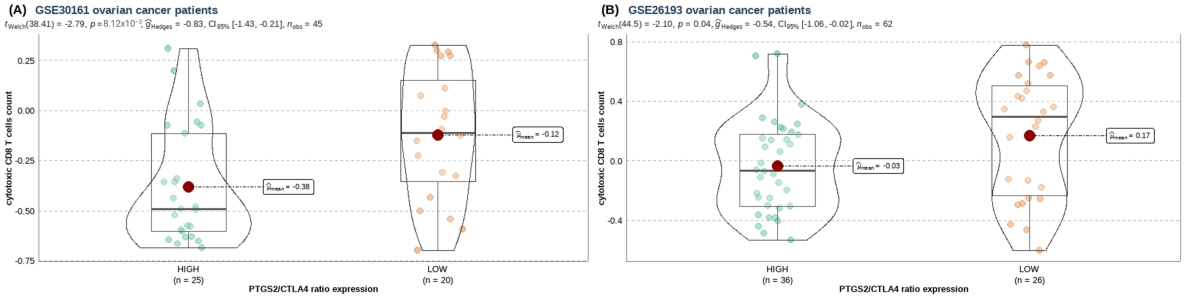

Figure S12. Comparison of GSVA score of cytotoxic CD8 T cells estimation from Nieto's signature for (A) GSE30161 and (B) GSE26193 ovarian cancer cohorts.

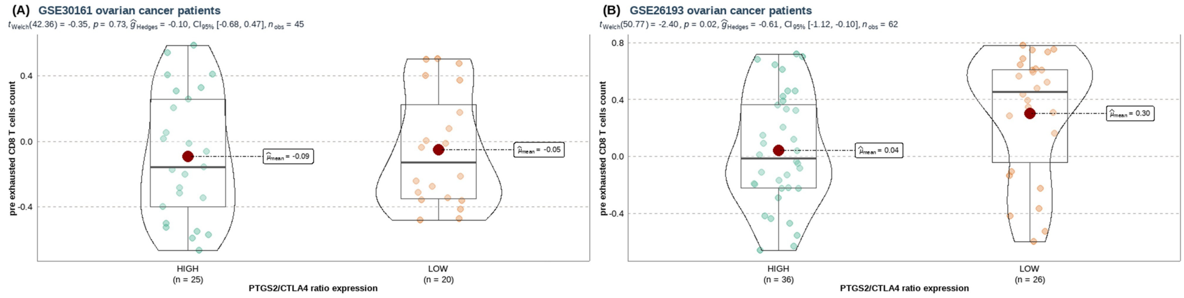

Figure S13. Comparison of GSVA score of pre-exhausted CD8 T cells estimation from Nieto's signature for (A) GSE30161 and (B) GSE26193 ovarian cancer cohorts.

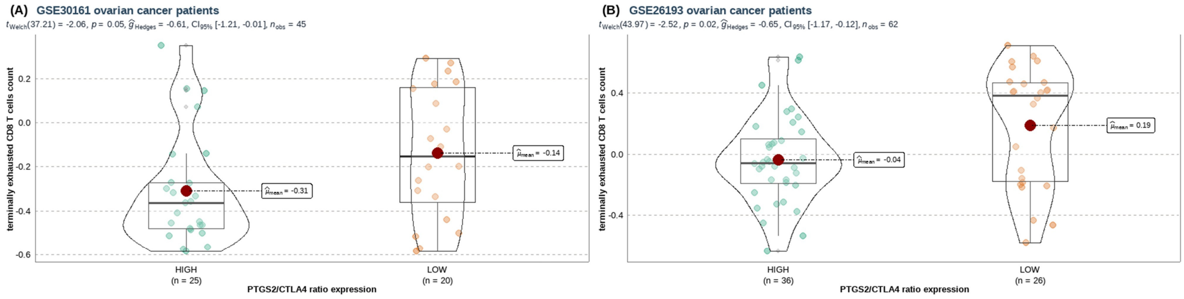

Figure S14. Comparison of GSVA score of terminally exhausted CD8 T cells estimation from Nieto's signature for (A) GSE30161 and (B) GSE26193 ovarian cancer cohorts.

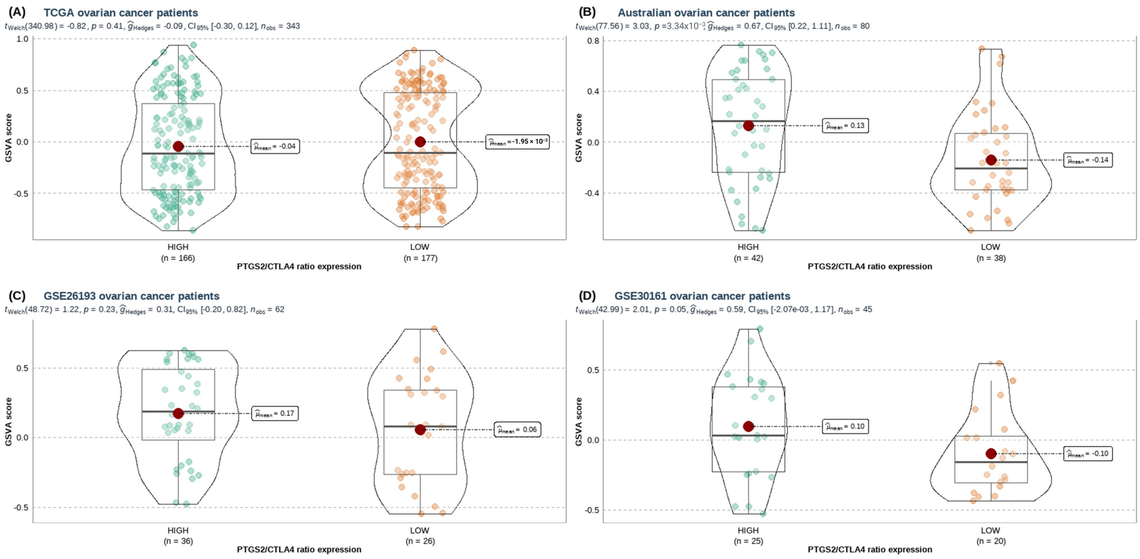

Figure S15. *PTGS2* signature (*PTGER2*, *PTGER4*, *PTGIS*, *PTGES* genes) GSVA enrichment comparison in (A) TCGA-GDC, (B) AOCS, (C) GSE26193, (D) GSE30161 ovarian patients' cohorts. Welch's *t*-test was performed through the "ggstatsplot" R package.

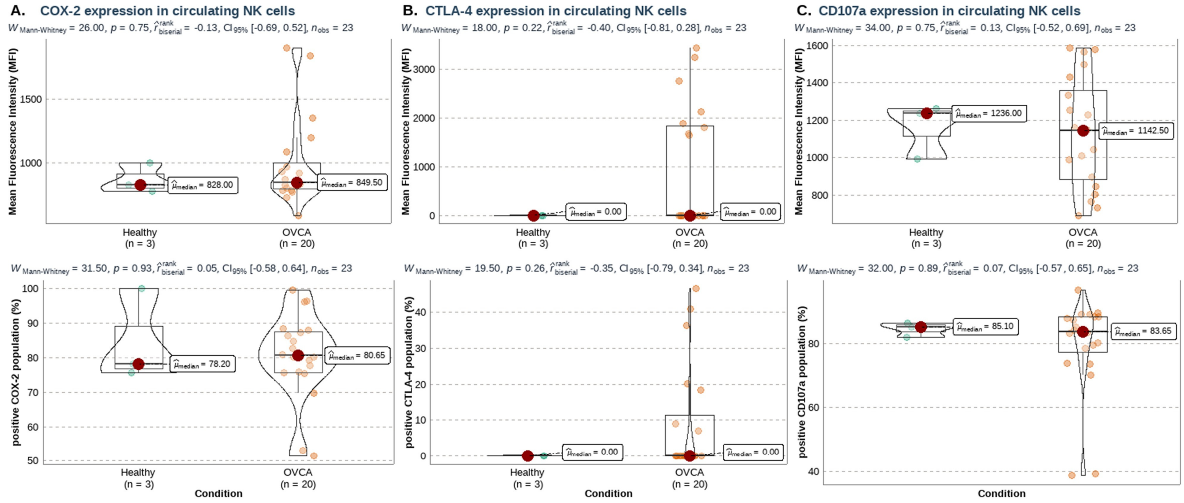

Figure S16. Comparison of Mean Fluorescence Intensity (MFI) and positive population (%) of (A) COX-2, (B) CTLA-4, and (C) CD107a expression between healthy donors and HGSOC patients. (OVCA, ovarian cancer).
